# Health Literacy and Associated Factors Among Military Personnel: A Cross-Sectional Study in Lithuania

**DOI:** 10.3390/healthcare14010103

**Published:** 2026-01-01

**Authors:** Saulius Sukys, Kristina Motiejunaite

**Affiliations:** Department of Physical and Social Education, Lithuanian Sports University, LT-44221 Kaunas, Lithuania; kristina.motiejunaite@lsu.lt

**Keywords:** armed forces, military personnel, health literacy, digital health literacy

## Abstract

Background: Health literacy is increasingly recognized as an essential determinant of health, readiness, and safety in the military, especially as health systems become more digitalized. However, evidence on general and digital health literacy in the armed forces remains limited. This study examined levels of general health literacy and digital health literacy among Lithuanian soldiers and explored their associations with sociodemographic, service-related, and health characteristics. Methods: A cross-sectional survey was conducted among 603 military personnel serving in the national armed forces. General and digital health literacy were measured with HLS19-Q12 and HLS19-DIGI. Data on sociodemographic and military characteristics, self-rated health, and self-reported long-term illnesses were collected. Descriptive statistics, correlation analyses, and multivariable regression models were used to analyze the data. Results: The sample was predominantly male (81.9%) with a mean age of 39.08 years (SD = 8.89). The mean general health literacy score was 80.1 (SD = 19.17), whereas the mean digital health literacy score was 67.81 (SD = 30.05). Overall, 45.0% of soldiers had excellent general health literacy, and 12.0% had inadequate general health literacy; 42.1% had excellent digital health literacy, and 35% had inadequate digital health literacy. Higher levels of health literacy were positively associated with better self-rated health and social status. No statistically significant associations were found between health literacy and gender, age, education, length of service, type of military service, and self-reported long-term health complaints. Conclusions: Military personnel in this study displayed relatively high general health literacy, yet digital health literacy was lower and more unevenly distributed, indicating a potential vulnerability for health outcomes as access to information, communication, and care increasingly relies on digital platforms. Given the cross-sectional design, causal inferences cannot be drawn. Military health services may build on existing health literacy strengths while considering strategies to address digital health literacy gaps (e.g., targeted training, tailored support, and user-friendly digital solutions, including service design), acknowledging that feasibility and implementation depend on organizational context and resources.

## 1. Introduction

Soldiers constitute a strategically significant yet vulnerable population whose health and functional capacity underpin national security and operational readiness. Research on military populations in Europe and beyond has largely focused on physical fitness and health conditions linked to performance and medical discharge (e.g., injuries, obesity, and cardiometabolic risk). Systematic reviews and empirical investigations demonstrate substantial variability in conscripts’ fitness levels and consistently link fitness and lifestyle behaviors with service outcomes [1,2,3,4].

A parallel literature addresses mental health and psychosocial determinants, including perceived and physiological stress [5,6,7,8], suicidal ideation [9,10], alcohol use [9,10], attitudes toward psychological help-seeking [11,12], and broader social and organizational factors shaping well-being in service [9,10,13,14]. While many military personnel report satisfactory health [15,16], a meaningful subgroup experiences lifestyle risk behaviors, mental health challenges, and adverse social conditions [17,18]. Despite this broad evidence base, comparatively little attention has been paid to foundational determinants such as health literacy, which may influence how soldiers interpret health information, navigate health services, and translate knowledge into health-related decisions and behaviors within the constraints of military life.

Health literacy constitutes a foundational determinant of health and plays a central role in promoting health equity. This concept refers to the knowledge, motivation, and skills required to access, comprehend, evaluate, and apply health information across contexts of care, disease prevention, and health promotion [19]. Large-scale population surveys across Europe indicate that limited health literacy remains widespread, with marked disparities observed between countries and social strata [20,21,22]. Lower levels of health literacy are particularly common among individuals with limited education, lower income, and disadvantaged social conditions, and have been associated with poorer self-rated health, reduced participation in preventive health behaviors, less effective management of chronic conditions, higher hospitalization rates, and elevated healthcare expenditures [20,21,22].

In tandem with traditional health literacy, digital and eHealth literacy have become increasingly important as health systems and communication environments are progressively digitalized. Digital health literacy encompasses the ability to seek, find, critically appraise, and apply health information from electronic sources to address health-related challenges [23]. Inadequate digital health literacy may exacerbate existing social disparities, constrain access to health services, and hinder effective self-management, especially as digital platforms, mobile applications, and online health information become more central to care delivery. Recent European initiatives, including the HLS19 European Health Literacy Survey and the IDEAHL project, highlight that improving both general and digital health literacy has become a priority for European health policy, particularly in relation to health promotion and reducing social and digital inequalities [21,24,25].

Evidence on health literacy among military populations remains limited, but emerging studies suggest that it may be an essential determinant of soldiers’ health and behavior. Several studies on health literacy have been conducted among military personnel in different countries. A study in China assessed the level of health literacy and its influencing factors among military personnel [26]. The results revealed that overall health literacy was relatively low, especially in the areas of lifestyle behavior and disease prevention. Another study involving military college students found that only 21.05% of participants had an adequate level of health literacy [27]. A third study investigated health literacy among military healthcare providers and showed that many participants lacked skills in maintaining health and preventing health problems [28]. Health literacy has also been studied among U.S. military personnel, where it was found that 99% of soldiers had adequate health literacy skills [29]. More recently, personal health literacy among U.S. Air Force personnel was examined, with a focus on its relationship with self-reported fitness activity, health-related quality of life, and health behavior tracking practices [30]. A study conducted among South African Navy personnel found that participants had a higher-than-average level of health literacy [31]. These studies reveal quite different data on the health literacy of soldiers.

Evidence on health literacy among European military populations is scarce. A recent cross-sectional survey of 695 Spanish Army personnel found that the mean general health literacy index, measured using the HLS-EU-Q47, fell within the sufficient range. However, health literacy was more strongly associated with formative and experiential factors—such as participation in soft-skills training, professional internships, and work in multidisciplinary teams—than with formal education or foreign language proficiency [32]. Another study among Spanish Army personnel reported that 37.9% of participants demonstrated excellent health literacy [33]. In contrast, a large study of Norwegian conscripts using the LS-Q12 found that 43% had inadequate health literacy. Those with lower health literacy more frequently reported poorer diet quality, worse oral health, and less favorable health behaviors, particularly in demanding contexts such as field exercises [18]. These findings suggest that health literacy among soldiers cannot be assumed to be uniformly high, may be shaped by the military training and working environment, and is linked to important health-related behaviors. However, data from Central and Eastern Europe remains limited. Even less is known about digital health literacy among military personnel worldwide. One step in this field was the validation of the eHealth Literacy Scale among U.S. military service personnel [34]. Additionally, a study of Royal Thai Army personnel using the eHealth Literacy Scale found that 92.3% of participants had adequate eHealth literacy [35]. Despite this, there is still limited knowledge regarding both general and digital health literacy among soldiers, particularly regarding their distribution across sociodemographic and service-related characteristics and their relationship with long-term health complaints, especially in countries such as Lithuania.

In summary, soldiers represent a highly relevant yet under-researched population in health literacy studies and health promotion policy both within individual countries and across Europe. Comprehensive data on the distribution of general and digital health literacy, and on how these domains are linked to sociodemographic and service-related characteristics, as well as long-term health outcomes, remain lacking.

In light of these gaps, the present study aimed to assess the distribution of general and digital health literacy and to examine their associations with sociodemographic, service-related, and health-related characteristics, including persistent health complaints, among Lithuanian military personnel.

Because empirical studies on soldiers’ health literacy, particularly digital health literacy, remain limited and existing findings are inconsistent, this study did not propose a priori hypotheses regarding soldiers’ health literacy levels or their associations with other variables.

## 2. Materials and Methods

### 2.1. Study Design, Setting, and Participants

In this cross-sectional study, the target population consisted of professional military service personnel in Lithuania. Conscripts were excluded from the study, and no other inclusion or exclusion criteria were applied. Because accurate data on the total number of professional soldiers were limited, the required sample size was calculated using the single population proportion formula: *n* = *Z*^2^ × *p*(1 − *p*)/*d*^2^, where *Z* = 1.96, *p* = 0.5, and *d* = 0.05.

Based on this calculation, the minimum required sample size was 384 participants. However, because the Lithuanian Armed Forces consist of soldiers and officers of different genders, ages, and military ranks, the study was not restricted to this minimum sample size. In total, 615 individuals participated in the survey, and data from 603 respondents were included in the final analysis.

Prior to data collection, ethical approval was obtained from the University Ethics Committee. In addition, since the study involved a sensitive population, permission to conduct the survey was granted by the national military commander.

Data collection was carried out via an anonymous online survey from March to May 2025. Invitation emails containing information about the study were distributed to personnel serving in different branches of the armed forces (e.g., land, air, and naval forces), as well as to military personnel working in the central military headquarters. Participants received information about the aim of the study and procedures implemented to ensure anonymity and confidentiality. They were informed that participation was voluntary and that they had the right to refuse participation or discontinue the survey at any point without any negative consequences. After providing electronic informed consent, participants were automatically redirected to the online questionnaire. The study, therefore, used a voluntary, non-probability sampling approach. As a result, some degree of self-selection cannot be excluded.

### 2.2. Study Measures

#### 2.2.1. Dependent Variables

In this study, general health literacy and digital health literacy were selected as dependent variables. 

General health literacy was measured by the HLS19-Q12 instrument. This 12-item questionnaire was developed on the basis of the European Health Literacy Survey Questionnaire with 47 items (HLS19-Q47) to measure comprehensive, general health literacy [21,22]. Previous cross-cultural studies showed adequate internal consistency (an average Cronbach’s alpha of 0.78) of this questionnaire [21,22]. This questionnaire was validated in Lithuania and also showed good internal consistency (α = 0.73 and ω = 0.75) [36]. This questionnaire starts with the following statement: “It is not always easy to get understandable, reliable, and useful information on health-related topics. With the following questions, we would like to find out which tasks related to handling health information are more or less easy or difficult. On a scale from very easy to very difficult, how easy would you say it is …” By answering all 12 items, participants had to choose one of four responses: 4 “Very easy”; 3 “Easy”; 2 “Difficult”; and 1 “Very difficult”. By analyzing the data, we calculated the overall score for HLS19-Q12 as a percentage (ranging from 0 to 100) and four levels of health literacy (excellent, sufficient, problematic, and inadequate). Following Pelikan et al. [22], the 0–100 index score was categorized using established cut points: inadequate (≤50), problematic (>50–66.67), sufficient (>66.67–83.33), and excellent (>83.33). 

Digital health literacy was measured by the Digital Health Literacy Questionnaire (HLS19-DIGI). This questionnaire was developed as HLS19-Q12 by the Health Literacy Population Survey [21,37] and validated in Lithuania [36,38] and showed good internal consistency in cross-cultural studies (an average Cronbach’s alpha of 0.83 [24] and in Lithuania (α = 0.73 and ω = 0.87) [36]. The HLS19-DIGI consists of eight items and a questionnaire prefaced with the statement, “When you search online for information on health, how easy or difficult is it for you?” By answering the items, participants had to choose one of four options: 4 “Very easy”; 3 “Easy”; 2 “Difficult”; and 1 “Very difficult”. The overall digital health literacy score was calculated the same way as the general health literacy score, and the value ranges from 0 to 100, with higher values indicating higher health literacy. We also calculated digital health literacy levels using the following criteria: less than 50 points—inadequate; between 50 and 66.66 points—problematic; between 66.67 and 83.33 points—sufficient; and above 83.34 points—excellent [22,39].

#### 2.2.2. Independent Variables

In this study, the independent variables selected were socio-demographic factors, including age, gender (male and female), educational level (in data analysis categorized as secondary and higher), and marital status (in data analysis categorized as married and other). As participants were soldiers, socio-demographics also included military service experience (categorized ≤ 5 years, 6–10 years, 11–20 years, 21–30 years, and more than 30 years), military rank (in data analysis categorized as soldier/sergeant and officer), type of military forces soldier belongs (in data analysis categorized as land forces, air forces, navy forces, and other as an example soldiers from the country’s military headquarters). These variables were selected a priori as plausible determinants of health literacy because health literacy is shaped not only by individual resources but also by social and organizational context. In a military setting, marital status can proxy social support that may facilitate help-seeking and navigation of health information and services; rank may reflect differences in role demands, training, and access to institutional information channels; and longer service experience may increase cumulative exposure to military health education and to digital systems used for communication and care. Force type was included to account for potential variation in duty context and access to digital infrastructure, which may be particularly relevant for digital health literacy.

The study also included other variables such as self-rated health, self-perceived social status, and long-term health complaints. Self-rated health was measured on a five-point scale: excellent, good, moderate, poor, and very poor. Social status was measured on a 10-point (ladder with 10 rungs) scale [40,41], with 10 points meaning the highest social status. Long-term health complaints were measured by asking, “Do you have any long-term illnesses or health problems? Long-term means problems that have lasted or are expected to last for 6 months or more?” Response categories were “Yes, more than one”, “Yes, one”, and “No, I don’t have”.

### 2.3. Data Analysis

All data analysis was performed using IBM SPSS version 31 (IBM Corp., Armonk, NY, USA). Before the main analysis, data for normality were screened by evaluating skewness and kurtosis. Variables’ normality was confirmed if skewness and kurtosis values did not exceed ±2 [42]. Next, a descriptive analysis (frequencies, percentages, and means with standard deviations) of participant socio-demographics and health literacy variables was performed. Finally, the association between independent variables and general and digital health literacy (separately) scores was measured by multiple regression analyses. The dependent variables are the overall scores from 0 to 100. The independent variables were gender (male = 0, female = 1), age as a continuous variable, marital status (other = 0, married = 1), educational level (secondary = 0, higher = 1), military service experience (≤5 years = 0, 6–10 years = 1, 11–20 years = 2, 21–30 years = 3, more than 30 years = 4), military rank (soldier/sergeant = 0, officer = 1), type of military forces (land forces = 0, air forces = 1, navy forces = 2, other = 3), and long-term health complains (don’t have = 0, one = 1, more than one = 2). Self-rated health is an ordinal variable ranging from 1 = very poor to 5 = excellent, and social status is an ordinal variable ranging from 1 as the lowest to 10 as the highest status. Before regression analysis, assumptions of linearity, independence, and homoscedasticity were verified. Multicollinearity was assessed using variance inflation factors (VIF < 5). Multicollinearity was not detected for any of the study variables. Model fit was evaluated using adjusted R^2^ and the F-statistic. Standardized beta coefficients (β) and 95% confidence intervals were reported, with statistical significance set at *p* < 0.05.

## 3. Results

### 3.1. General Sample Characteristics

A total of 615 participants participated in this study. Since 12 (1.95%) participants did not complete the questionnaire, we analyzed the data of 603 soldiers. One-fifth of them were male (81.9%), and the mean age of participants was 39.08 years (SD = 8.89), ranging from 20 to 57 years. Table 1 shows the distribution of other social-demographic characteristics, such as age, marital status, service experience, military rank, type of military forces, education, and personal health, life evaluation, and long-term illnesses. 

### 3.2. General and Digital Health Literacy

The results of the study indicated that the mean general health literacy score among soldiers was 80.1 (SD = 19.17), whereas the mean digital health literacy score was 67.81 (SD = 30.05) (Table 2). A statistically significant correlation was observed between general and digital health literacy.

**Figure 1:**
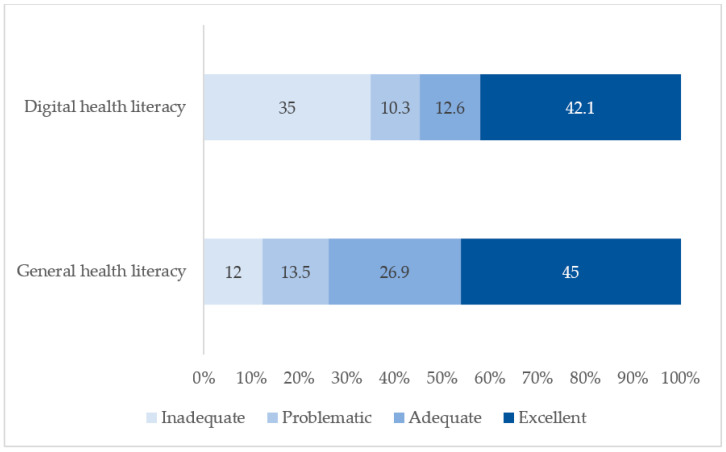
Distribution of general and digital health literacy levels.

Analysis of health literacy levels revealed that 45.0% of soldiers demonstrated an excellent level of general health literacy, while 42.1% exhibited an excellent level of digital health literacy (Figure 1). In contrast, inadequate general health literacy was identified in 12.0% of soldiers, whereas inadequate digital health literacy was observed in approximately one-third of the sample.

### 3.3. Factors Associated with General and Digital Health Literacy

The first multiple linear regression analysis examined the relationship between social demographics and general health literacy. Independent variables included gender, age, marital status, educational level, military service experience, military rank, type of military forces, and self-rated health, social status, and long-term illnesses. We found that the combination of these variables significantly predicted general health literacy (F = 9.84, *p* < 0.001) and explained 13% of the variance in general health literacy (Table 3). When evaluating individual independent variables, it was found that being married was significantly positively related to higher health literacy (β = 0.10, *p* = 0.02). Better-rated personal health (β = 0.20, *p* < 0.001) and higher social status (β = 0.23, *p* < 0.001) were also positively related to general health literacy.

The second regression analysis was performed with digital health literacy as the dependent variable using the same independent variables. Study results showed that the regression model was significant (*F* = 4.63, *p* < 0.001) and explained 6% of digital health literacy variance. As shown in Table 3, among all independent variables, self-rated health (β = 0.10, *p* = 0.04) and social status (β = 0.19, *p* < 0.001) were significantly positively related to digital health literacy.

## 4. Discussion

This study examined general and digital health literacy among Lithuanian military personnel and explored how these constructs are associated with sociodemographic, service-related, and health-related characteristics. Overall, the findings showed relatively high mean levels of general health literacy and comparatively lower digital health literacy, with a strong positive correlation between the two. Almost half of the soldiers demonstrated excellent health literacy, and more than two-fifths had excellent digital health literacy. At the same time, inadequate health literacy was identified in one in eight soldiers, whereas inadequate digital health literacy was observed in roughly one-third of the sample.

The relatively high level of general health literacy observed in this study is consistent with findings from military populations in other countries [29,31]. A European study reported similar, although somewhat lower levels of health literacy among soldiers [33]. However, cross-study comparisons should be interpreted with caution because the studies used different measurement instruments, such as the S-TOFHLA, REALM, HLQ, and the Chinese Citizen Health Literacy Questionnaire. Notably, none of the previous studies in military populations have employed the HLS19-Q12 instrument used in the present research. Comparing digital health literacy is even more challenging due to the limited availability of relevant data. In the few existing studies, such as research among Thai soldiers [35], Lithuanian soldiers appear to display lower levels of digital health literacy. 

It is therefore important to contextualize soldiers’ health literacy by comparing it with that of the general population. However, national data in Lithuania remain scarce. A comparative study of Lithuanian and Latvian populations showed that 51% of Lithuanians had problematic general health literacy, 24% had sufficient health literacy, and only 3% were classified as having an excellent level [43]. In comparison, the results of the present study suggest that soldiers exhibit higher general health literacy than the adult population overall. Due to the lack of national data on digital health literacy, analyses in this domain remain limited and must rely on indirect comparisons with data from other European countries, particularly those using the same measurement tool. Results from 13 countries showed that the mean score across all countries was 62.3 [24], which is very similar to that found in our study with soldiers.

Regression analyses indicated that combinations of sociodemographic, service-related, and health variables significantly predicted both general and digital health literacy, although the explained variance was modest (adjusted R^2^ = 0.13 and 0.06, respectively). Among the individual predictors, better self-rated health and higher subjective social status were consistently associated with higher general and digital health literacy, and being married was also positively related to general health literacy. These associations are consistent with previous research showing that lower health literacy is linked to poorer self-rated health [44,45] and that subjective social status and health literacy jointly contribute to health and mental health outcomes [46,47]. In contrast, gender, age, education, years of military experience, rank, type of military forces, and long-term health complaints were not significantly associated with either form of health literacy. Taken together, these findings suggest that, within this predominantly male, working-age military cohort, soldiers’ perceptions of their own health and social standing may be more important for health literacy than basic sociodemographic or service-related characteristics, and that additional unmeasured contextual and organizational factors are likely to play a role. The modest explained variance suggests that health literacy in military settings may be shaped by factors not captured in the present models. Conceptually, such influences can be viewed as organizational opportunity structures that shape exposure to health information, access to digital resources, and the perceived costs and benefits of engaging with services. This may be especially relevant for digital health literacy, which depends on connectivity, permitted device use, and the usability of institutional platforms. These may include unit- and base-level differences in digital infrastructure and connectivity, the availability and usability of military-specific digital health services (e.g., portals, e-record access, teleconsultation workflows), and differential exposure to health promotion or digital skills training. In addition, operational tempo, shift patterns, and limited time for health-related information seeking may influence both engagement with health information and the development of digital competencies. Finally, aspects of military culture—such as norms around self-reliance, perceptions of confidentiality, and attitudes toward help-seeking—may shape how service members interact with health information and services, potentially affecting observed associations.

Taken together, these patterns point to a mixed health literacy profile among soldiers: on the one hand, relatively high levels of general health literacy, and on the other hand, noticeably lower and more uneven levels of digital health literacy, with a sizeable subgroup scoring in the inadequate range. Recent European data using the HLS19-DIGI instrument similarly show substantial variation in digital health literacy across population groups and countries, underscoring that digital health literacy is often more fragile and unequally distributed than general health literacy [24]. This discrepancy is particularly important in the context of the ongoing digitalization of health systems, where access to information, communication with health professionals, and use of self-management tools increasingly depend on digital platforms [48]. Digital health literacy may be more sensitive than general health literacy to opportunities for routine digital engagement, access to stable connectivity, and organizational constraints on device use, which can vary across military units and duty contexts. For soldiers, digital competencies are relevant not only for routine healthcare but also in operational settings, for example, when using telehealth services, electronic records, or remote consultation systems that are increasingly used in military health services [49,50]. The lower levels of digital health literacy observed in our sample, therefore, suggest a potential vulnerability: soldiers with limited digital health literacy may have more difficulty locating trustworthy online information, judging its credibility, and applying it in complex situations, even if their general health literacy is relatively high. In increasingly digitalized military health systems, such gaps could hinder timely access to reliable information and digital services and may therefore have downstream implications for medical readiness and, consequently, operational readiness. Addressing this gap may require targeted training and user-friendly digital solutions that explicitly support soldiers with lower digital health literacy rather than assuming that general health literacy automatically translates into digital competence, as also suggested by telehealth initiatives that aim to bridge digital divides in other uniformed and veteran populations [51,52].

This study suggests that general health literacy is higher among married military personnel. Although marital status was not evaluated in all previous studies of soldiers, those that assessed this sociodemographic factor did not find a significant association with health literacy [29]. Our results also showed that both general and digital health literacy were related to soldiers’ subjective social status, suggesting that those who perceived their personal life as better had higher levels of health literacy. These findings are consistent with population-based studies demonstrating that social status is one of the factors most strongly associated with health literacy [24], including research conducted in the Lithuanian population that revealed similar associations [43]. In the context of our study, it is also worth noting that research on military school students has shown that their health literacy is positively associated with higher family socioeconomic status [27]. Similarly, health literacy among military healthcare providers has been found to be positively related to higher socioeconomic status [28]. Taken together, these findings underscore the importance of social position and family resources as potential determinants of health literacy in military settings, and suggest that efforts to strengthen health literacy may also need to address underlying social and economic inequalities.

In our study, higher levels of health literacy were positively associated with better self-rated health, which is consistent with previous findings in military populations [18,27]. Studies among Chinese military academy students [27] and active-duty Air Force personnel [30] likewise show that individuals with higher health literacy tend to evaluate their health more favorably. However, we did not observe a significant association between health literacy and self-reported long-term health complaints. This result may reflect several characteristics of our sample and context. First, soldiers constitute an occupationally selected, working-age population in which long-term health complaints may be more strongly related to length of service, specific work- and service-related exposures, or biological risk factors than to individual competencies such as health literacy. Second, universal and readily accessible military healthcare may attenuate some of the adverse consequences that are typically associated with low health literacy in the general population, thereby reducing observable differences in chronic morbidity [53,54]. Finally, our cross-sectional design and the relatively broad measure of “long-term complaints” may not capture the more complex pathways through which health literacy can influence the management, severity, or course of chronic conditions over time.

We also did not find statistically significant associations between health literacy and gender, age, education, length of service, or type of military service in our sample. Similar patterns have been reported in other military studies, where health literacy levels did not differ systematically by gender or age, and mode of service showed no consistent gradient [27,30]. When compared with general adult populations, the picture is more heterogeneous. Population-based surveys in Europe, including the HLS19 study, typically show social gradients in health literacy, with lower levels more common among older adults and those in less advantaged socioeconomic positions [21]. For example, a recent Baltic study found no association between health literacy and gender, but a clear negative association with age in Latvian and Lithuanian adults [43]. Taken together, these findings suggest that recruitment criteria, medical fitness standards, and standardized training in the military may attenuate some of the sociodemographic inequalities in health literacy that are observed in the general population, even though differences may still emerge when more specific competencies—such as digital health literacy—are considered [24].

Taken together, our findings provide a nuanced portrait of health literacy in a contemporary military context. While overall health literacy among military personnel appears to be relatively high and is positively associated with their self-rated health, digital health literacy is lower and more unevenly distributed, with a significant proportion of service members at risk of exclusion in increasingly digitalized health systems. At the same time, the absence of pronounced social and demographic gradients suggests that recruitment criteria, medical fitness standards, and standardized training may mitigate some of the inequalities typically observed in the general population. This combination of strengths and vulnerabilities underscores the imperative for military health services to leverage existing health literacy resources while also explicitly addressing gaps in digital health literacy through dedicated training, support, and the development of user-friendly digital solutions.

### Strengths and Limitations

This study has several strengths. First, to our knowledge, it is one of the first studies to provide a detailed description of both general and digital health literacy among military personnel, using the same conceptual and measurement framework that has been applied in large population-based surveys. The use of validated instruments for assessing health literacy and digital health literacy enhances the comparability of our findings with international evidence and strengthens the internal validity of the measurements. Second, the sample included soldiers across a broad working-age range and with diverse service characteristics (different ranks, branches of service, and length of service), allowing us to explore associations with sociodemographic and service-related factors rather than focusing on a single subgroup. Third, the study simultaneously examined general and digital health literacy in relation to self-rated health and self-reported long-term health complaints, thereby providing a more nuanced picture of how different dimensions of health literacy may relate to health in a military context.

However, several limitations should be considered when interpreting the results. The cross-sectional design precludes any causal inference regarding the direction of associations between health literacy, digital health literacy, and health outcomes; longitudinal studies are needed to clarify whether higher health literacy leads to better health, or whether individuals in better health are more likely to report higher health literacy. All data were based on self-reports, which may be subject to recall bias and social desirability bias, particularly in a military context where norms around fitness and readiness are strong. In addition, as participation was voluntary, the sample may be subject to self-selection; for example, service members with greater interest in health-related topics, higher health literacy, or higher confidence using digital tools may have been more likely to participate. The interpretation of associations with long-term health complaints is further constrained by the relatively low prevalence of self-reported chronic conditions and the high proportion of participants rating their health as good or excellent, which may have reduced statistical power to detect differences between groups. Finally, some covariates were dichotomized (e.g., education, marital status), which may have reduced variability and explanatory power.

The generalizability of our findings is also limited. The study was conducted within a single national military context, with its specific recruitment criteria, organizational culture, and healthcare system. Moreover, although we included soldiers from different ranks and branches of service, our sample was predominantly male (81,9%), and the majority had higher education (74.3%), which may limit the applicability of the results to armed forces with different gender or educational profiles. While a male-dominated sex distribution is expected in this occupational setting, the findings may not generalize to forces with substantially different gender or educational compositions and should not be interpreted as a benchmark for more diverse military populations. We also relied on a relatively broad measure of long-term health complaints and did not include objective clinical indicators or performance-based measures of health literacy or digital skills, which might have captured additional aspects of vulnerability. Finally, while the digital health literacy instrument allowed us to assess key competencies relevant to navigating online health information, it did not specifically address the usability or accessibility of concrete military digital health platforms, which may involve additional training and system-level factors beyond individual literacy. Together, these limitations suggest that the results should be interpreted with appropriate caution and highlight the need for further research, including longitudinal and intervention studies, in diverse military settings.

## 5. Conclusions

This study provides new evidence on general and digital health literacy among military personnel in a contemporary military setting. Overall, general health literacy levels were relatively high and positively associated with self-rated health, suggesting that many soldiers possess the competencies needed to access, understand, and use health information in everyday life. In contrast, digital health literacy was lower and more unevenly distributed, with a sizeable subgroup scoring in the inadequate range. This imbalance indicates a potential vulnerability in an era when health systems and military health services are becoming increasingly digitalized.

The absence of marked sociodemographic gradients in health literacy by gender, age, education, length of service, or type of military service suggests that recruitment criteria and standardized training may attenuate some of the inequalities observed in general adult populations. At the same time, our findings underline that digital health literacy cannot be assumed to mirror general health literacy, even in a highly organized and training-intensive environment such as the armed forces.

For military health services and policymakers, these findings suggest that digital health literacy merits explicit attention alongside efforts to maintain and strengthen general health literacy. At the individual level, potential approaches may include targeted training and tailored user support for service members with lower digital health literacy. At the system level, these implications also point to the importance of designing and refining military digital health platforms and workflows (e.g., portals, e-record access, and teleconsultation pathways) to be usable and accessible for users with varying digital competencies. However, the extent to which such measures can be implemented will depend on local infrastructure, organizational priorities, and available resources. Future research should, therefore, examine the feasibility, acceptability, and effectiveness of specific interventions in military settings, for example, through pilot programs and implementation-focused evaluation. 

## Figures and Tables

**Table 1:** Descriptive statistics of sociodemographic characteristics of the study participants.

Variable	Categories	N	%/M (SD)
Age		603	39.08 (8.89)
Genders	Females	109	18.1%
	Males	494	81.9%
Education	Secondary education	155	25.7%
	Higher education	448	74.3%
Marital status	Married	436	72.3%
	Other	167	27.7%
Military service experience			15.70 (9.30)
Military service experience groups	≤5	69	11.5%
	6–10	145	24.1%
	11–20	142	23.6%
	21–30	197	32.7%
	31+	26	8.1%
Military rank	Soldier/sergeant	366	60.7%
	Officer	237	39.3%
Type of military forces	Land forces	388	64.3%
	Air forces	74	12.3%
	Navy forces	39	6.5%
	Other	102	16.9%
Self-rated health	Excellent	130	21.6%
	Good	343	56.9%
	Moderate	121	20.1%
	Poor	8	1.3%
	Very poor	1	0.2%
Social status		603	7.31 (1.43)
Long-term illnesses	Don’t have	354	58.7%
	One	168	27.9%
	More than one	81	13.4%

Note: N: number of participants; %: percentage; M: mean; SD: standard deviation.

**Table 2:** Descriptive statistics of general and digital health literacy.

	Mean	Standard Deviation	α	Skewness/Kurtosis	Correlation
GHL	80.1	19.17	0.81	−1.02/0.45	
DHL	67.81	30.05	0.84	−0.56/0.82	0.63 **

Note. GHL—general health literacy, DHL—digital health literacy. ** *p* < 0.01.

**Table 3:** Multiple regression analysis of influencing factors associated with the health literacy of soldiers.

	General Health Literacy				Digital Health Literacy			
	β	*p*	95% CI		β	*p*	95% CI	
			LL	UL			LL	UL
Gender	−0.02	0.68	−4.77	3.11	0.01	0.95	−6.12	6.53
Age	−0.05	0.56	−0.45	0.24	−0.07	0.43	−0.75	0.33
Education	−0.03	0.55	−5.01	2.72	−0.01	0.76	−7.20	5.23
Marital status	0.10	0.02	0.74	7.96	0.01	0.86	−5.30	6.33
Experience in years	0.02	0.86	−2.43	2.91	0.03	0.76	−3.57	4.86
Military rank	0.02	0.77	−2.93	3.97	−0.04	0.39	−8.03	3.11
Type of military forces	−0.01	0.86	−1.37	1.45	0.01	0.93	−1.94	2.13
Social status	0.23	<0.001	2.00	4.20	0.19	<0.001	2.15	5.68
Self-rated health	0.20	<0.001	2.91	7.94	0.10	0.04	0.04	8.15
Long-term health complaints	0.04	0.36	−1.25	3.42	0.07	0.13	−0.85	6.66
	AdjR^2^ = 0.13, F = 9.84, *p* < 0.001				AdjR^2^ = 0.06, F = 4.63, *p* < 0.001			

Note. β: standardized coefficients, 95% CI: 95% Confidence Interval for β, LL: lower limit, UL: upper limit.

## Data Availability

The data presented in this study are available on request from the corresponding author. The data are not publicly available due to privacy and ethical restrictions.
